# Diffusion Mechanism of Cinnamon Essential Oils Release from Calcium Alginate Based Controlled Release Films in Contact with Food Simulating Solvent

**DOI:** 10.3390/ma13245679

**Published:** 2020-12-12

**Authors:** Xi Chen, Li-Xin Lu, Wei-Rong Yao, Liao Pan

**Affiliations:** 1 Department of Packaging Engineering, Jiangnan University, Wuxi 214122, China; 8201901012@jiangnan.edu.cn (X.C.); breath860101@aliyun.com (L.P.); 2 Key Laboratory of Advanced Food Manufacturing Equipment and Technology of Jiangsu Province, Jiangnan University, Wuxi 214122, China; 3 State Key Laboratory of Food Science and Technology, Jiangnan University, Wuxi 214122, China; yaoweirongcn@jiangnan.edu.cn

**Keywords:** diffusion mechanism, controlled release, bio-based film, solvent, cinnamon essential oils, calcium alginate

## Abstract

Calcium alginate based controlled release films with moderate mechanical properties were fabricated in this paper. The diffusion mechanism of these films contacting food simulating solvent (FSS) was explored in some detail. With the increase of glycerol content, the diffusion coefficient (*D*) values of cinnamon essential oils (CEOs) diffusing to ethanol first increased slowly (0.3–0.6 mL), then vigorously (0.6–0.9 mL), and then mildly (0.9–1.2 mL). The *D* values of the CEOs diffused to water are all in the order of magnitude of 10^−10^ cm^2^/s. The *D* values of CEOs diffused from films EG3 and EGC1 to aqueous ethanol altered enormously at a small moisture percentage (*w* = 0.3), then continuously varied vigorously, and at last altered mildly in the range of *w* = 0.3–1. All the results above indicate that, considering the FSS, the diffusion ability of molecules is jointly determined by the size and distribution of free volume in the system (polymer + diffusive substance + solvents), the intermolecular interaction, and the partition coefficient of the solvents. In addition, several pairs of *D* values, such as *D*_EG_ and *D*_GA_, are very close to each other, indicating that different kinds of interactions between different groups may have the same effect on the diffusion ability of molecules. The correlation between *D*_1_ and *D*_2_ indicates that polymeric emulsifier chains also exist in the polymer-rich layer. All the findings and analysis could provide the theoretical basis and data support for further molecular dynamic simulation and could guide the design of controlled release food packaging for food protection.

## 1. Introduction

Bio-based controlled release food packaging has become a research hotspot in the field of food protection because of its advantages of safety, environmental friendliness, better fresh-keeping effect, and longer shelf life [1,2,3]. However, most studies focus on the methods and effects of the controlled release technique, and as for the mechanism, it is only mentioned very briefly, with short descriptions such as “the tortuosity of release path is different” [4]. How the active substance is released through bio-based film from a perspective of kinetic molecular theory is seldom clarified. Moreover, the mechanism of active substance release through bio-based film can become more complicated than that through regular plastic packaging films. This might be because of the existence of multiple polar functional groups, such as –OH, –NH_2_, –COOH, and an abundant hydrogen bond that not only makes a fine quantity of molecular interactions very difficult, but it also makes the film highly sensitive to certain food ingredients, such as H_2_O [5]. Different ingredients of food can permeate the film leading to more kinds of molecular interactions, thus further complicating the microscopic release mechanism of active substances. However, interactions such as swelling led by moisture is sometimes beneficial, which, for instance, acts as a trigger for controlled release [6]. Therefore, more studies are needed to understand the microscopic mechanism of active substance release through bio-based film.

The foundation theory to describe active substance release through bio-based film is the diffusion theory of molecules. The diffusion phenomenon is obtained by the random thermal motion of molecules above critical temperature from the perspective of kinetic molecular theory. Therefore, Einstein’s theoretical formula can be presented to express the diffusion ability of a single molecule (Equation (1)) and many molecules (Equation (2)) in an ideal gas/liquid by 3-dimensional random walking [7], where the diffusion ability is characterized by the diffusion coefficient (*D*). It can be seen from Equations (1) and (2) that the average moving step length and the time required to move in such a step length are the two major factors affecting the diffusion ability of molecules.

(1) $$\langle {\left|r-r_{0}\right|}^{2}\rangle =6Dt$$

(2) $$D=\frac{1}{6N}\underset{t\to \infty }{\lim}\frac{d}{dt}\sum_{i=1}^{N}\langle {\left|r_{i}\left(t\right)-r_{0}\right|}^{2}\rangle$$

where *r* is the position of molecule at time *t*, *r*_0_ is the original position of molecular at time *t* = 0, *D* is the diffusion coefficient of molecule, *t* is the diffuse time, *N* is the total amount of molecules, *i* is the number of molecules, and *r_i_*(*t*) is the position of the molecule of number *i* at time *t*. Because the actual time interval to test *D* is far longer than that of molecular translational motion, the limit of *t* tends toward infinity.

Compared with the strong diffusion ability of molecules in gas/liquid (molecules can move relatively freely and rapidly with a large average step size), the diffusion ability of molecules in polymers is much weaker. This might be due to the large molecular volume, dense packing mode, and small motion amplitude of the polymer chain, which leaves very little space (free volume) for the free movement of diffusion molecules. Diffusion molecules act like being trapped by entangled chains in the gap between chains, and it can be difficult to move them [8]. Most of the random thermal motion of the molecules constantly rebounds by the gap wall, equivalent to standing still without displacement. This process takes a long time, in the order of 0.1 nanoseconds. Only when the free volume between the chains redistributes to form a channel between holes can molecules jump to the adjacent holes and make a move step. This process takes place very briefly [9], in the order of a picosecond, which is far less than the residence time in the holes, so it is often ignored in the count of total moving time. Therefore, the path of molecular diffusion consists of every transition displacement. The interval between two transitions, or the time trapped in the hole, is equivalent to the time required for one displacement. Einstein’s theoretical formula is also applicable. The diffusion ability is directly determined by the mean squared displacement (MSD) per unit time. With a smaller MSD and longer time, the *D* value of the molecules diffused in polymers is significantly smaller than that of molecules diffused in gas/liquid.

In order to further describe the influence mechanism of polymers on molecular diffusion, the free volume theory [10] proposed that the transition displacement and transition interval of molecules diffused in polymers depend on three points as follows: (1) the total amount of free volume or the sum volume of all the holes; (2) the distribution of free volume, such as size and location of each hole, especially the distance between adjacent holes; and (3) the amount of energy required for the redistribution of free volume (the motion ability of chain segments in order to connect the holes).

However, some parameters in free volume theory rely on a “smart guess” [11]. In order to explain the diffusion mechanism of molecules in polymers from the first principle, the three key points of free volume theory should be revisited from the perspective of molecular dynamics. In a system with specified conditions, the total amount of free volume is determined by the chemical composition and bonding of all atoms, based on which the size and the distribution of free volume are determined by the configuration and conformation of the chain. Based on all the above (i.e., composition, bonding, configuration, and conformation), the energy required for free volume redistribution is determined by the force field or interaction between molecules.

Therefore, on the foundation of the molecular dynamics theory, the model of molecule diffusion in polymers can be constructed by computer simulation. Then, the *D* value and the microscopic mechanism affecting diffusion can be analyzed through the molecular dynamic simulation results. In general, at least 10 transitions have to be observed. However, the simulation time of the commonly used material studio (MS) method of molecular dynamics simulation for diffusion is 10 ns, which means that when the transition distance is 0.5 nm, *D* values less than 10^−7^ cm^2^/s cannot be calculated. However, with the regular transition distance being 0.5 nm [12], the *D* value of diffusion in packaging films are usually in the range of 10^−9^ to 10^−11^ cm^2^/s, which requires simulation time to increase to 1–100 μs, and the amount of calculation increases exponentially. Therefore, it is necessary to find other molecular dynamics methods and optimize the algorithm to make the calculation accurate and fast.

In addition, to get close to the real situation, not only the interaction between atoms in the polymer chain and interaction between diffusion molecules and polymer atoms should be considered, but the interaction among additives (such as plasticizers), properties of food ingredients (such as moisture), and other solvents that permeate the film should also be considered. The real environment for molecular diffusion is the force field consisting of interactions among all the atoms in these films.

In conclusion, with such a complicated force field that makes it hard to build fine molecular dynamics simulation currently, it is necessary to analyze the molecular diffusion mechanism based on experiments and existing theories. Previous research showed that complex biopolymeric emulsifiers significantly controlled the release of cinnamon essential oils (CEOs) from sodium alginate based films, and the films could be potentially applied as active packaging for the protection of the food inside [13]. Therefore, in this paper, calcium alginate based controlled release films are fabricated, and the effects of a plasticizer (glycerin) and food simulant solvent (FSS; including ethanol, water, and their mixture) on the diffusion of cinnamon essential oil (CEO) molecules through crosslinked calcium alginate based film are obtained through experimental research. Furthermore, the microscopic mechanism is analyzed based on molecular dynamics and free volume theories, which provide the theoretical basis and data support for later computer simulation.

## 2. Materials and Methods

### 2.1. Materials

Sodium alginate (SA, relative molecular weight *M_r_* = 216.12303, viscosity *η* ≥ 0.02 Pa s), gelatin (G, *M_r_* = 10,000–70,000, *η* ≥ 0.015 Pa s), gum acacia (A, *M_r_* = 262.64858, *η* = 0.06–0.13 Pa s), carboxymethylcellulose sodium (C, *M_r_* = 263.1976, *η =* 0.3–0.8 Pa s), cinnamon essential oil (CEO, density *ρ* = 1.045–1.070 g/mL), glycerol (Gly), calcium chloride (CaCl_2_), and absolute ethanol were all purchased from Sinopharm Chemical Reagent Co., Ltd. (Shanghai, China). All chemicals were of analytical grade. Ultrapure water was prepared by the Aquaplore ultra-pure water system (Shanghai Ultrapure Industrial Co., Ltd., Shanghai, China).

### 2.2. Preparation of Films

The o/w emulsions, film forming solutions (FFSs), and the films were prepared and stored according to the method described by Chen et al. [13]. All the formula and technique parameters were the same except for two changes. Firstly, the content of glycol altered as shown in the formulation in Table 1; secondly, after the films G, GA, and GC were obtained, each film was immersed in 25 mL of 2 wt% aqueous CaCl_2_ solution for 30 s to be externally crosslinked and then dried at room temperature.

### 2.3. Scanning Electron Microscopy (SEM)

SEM photos were obtained through a field emission Hitachi S-4800 SEM (Hitachi, Ltd., Tokyo, Japan) at a voltage of 2.0 kV. The film samples were attached to cylindrical copper stub by double-sided adhesive tape and pretreated with gold spraying.

### 2.4. Film Thickness and Mechanical Properties of Films

The average of five random measurements along the specimen using a micrometer (Liuling Instruments, Shanghai, China) was used to determine the film thickness. The tensile strength (*TS*) and the elongation (*E*) at the break of the crosslinked films were tested by a universal testing machine (LLOYD; AMETEK, Shanghai, China). The specific settings and pretreatment are the same as those in previous studies [13,14].

### 2.5. Swelling Properties

The mass of film samples in release studies at each sampling time were weighed and recorded. The swelling property of films are characterized by swelling rate (*s*) calculated with Equation (3) [15].

(3) $$s=\left(m_{t}-m_{0}\right)/m_{0}$$

where *m*_0_ is the mass of dry film, *m*_t_ is the mass of swelled film at time *t*, and thus (*m*_t_−*m*_0_) is the mass of adsorbed liquid at time *t*. In addition, when the swelling process reaches equilibrium at time *t*, then *s* is the swelling rate at equilibrium.

### 2.6. Release Studies

The one-way release studies were performed according to the method described by Chen et al. [13]. In brief, the one-way release exam was carried out using a self-designed one-way release instrument at 4 °C, with sampling at different time points. Although CEO is a mixture containing many ingredients, most of it (98.2%) is cinnamaldehyde. Therefore, the characteristic wavelength 286 nm for cinnamaldehyde detection by the UV spectrophotometer is also suitable for CEO. Hence, the concentration of CEO in FSS was detected by a UV-1800 Spectrophotometer (Shimadzu Corporation, Kyoto, Japan) at a wavelength of 286 nm and quantified through a linear regression analysis with a correlation coefficient (*R*^2^) of 0.9994. Specifically, FSS was chosen as absolute ethanol, with water proportion *w* = 0, 95% aqueous ethanol (*w* = 0.05), 70% aqueous ethanol (*w* = 0.3), 40% aqueous ethanol (*w* = 0.6), 5% aqueous ethanol (*w* = 0.95), and water (*w* = 1). Then based on the exam conditions, the *D* values of the CEO released from films to FSS and the corresponding theoretical values were calculated from the modeling of the release curve versus time according to the solution (Equation (4)) of Fick’s second law with MATLAB (MathWorks, Natick, MA, USA).

(4) $$\frac{M_{F,t}}{M_{F,\infty }}=1-\sum_{n=0}^{\infty }\frac{8}{{\left(2n+1\right)}^{2}\pi^{2}}\exp\left[-\frac{D{\left(2n+1\right)}^{2}\pi^{2}t}{4d_{P}{}^{2}}\right]$$

where *M_F,t_* is the amount of diffusing substance diffused at time *t*, *M_F,∞_* is the amount of diffusing substance diffused at equilibrium, *d_P_* is the film thickness, and *D* is the diffusion coefficient.

For the diffusion in multilayer complex film, there is a functional relationship (Equation (5)) between the total diffusion coefficient and the diffusion coefficient of each layer [16]:

(5) $$d_{P}/D_{P}=d_{P,1}/D_{P,1}+d_{P,2}/D_{P,2}+\cdots +d_{P,n}/D_{P,n}$$

where *d*_P_ is the total thickness of film; *D*_P_ is the diffusion coefficient of substance diffused through all the layers of film; *d*_P,1_, *d*_P,2_, …, *d*_P,n_ are thicknesses of each layer of film; and *D*_P,1_, *D*_P,2_, …, *D*_P,n_ are diffusion coefficient values of the substance diffused through each layer of film.

### 2.7. Statistical Analysis

An one-way analysis of variance was employed for statistical analysis with the SPSS computer program (SPSS Inc., version 22). The Tukey test was applied for the evaluation of differences in pairs of mean values with a confidence interval of 95%.

## 3. Results and Discussion

### 3.1. Two-Layer Structure of Film

**Figure 1:**
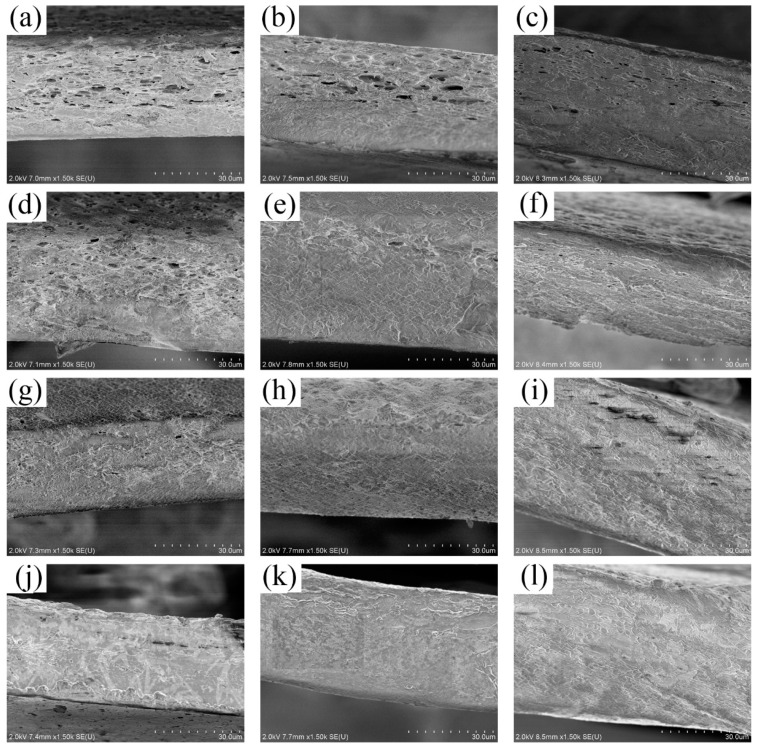
SEM images of the cross sections of films EG1–EG4 (**a**–**d**), EGA1–EGA4 (**e**–**h**), EGC1–EGC4 (**i**–**l**).

**Figure 2:**
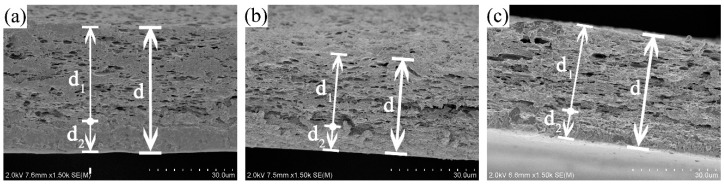
SEM images of the cross sections of films G (**a**), GA (**b**), and GC (**c**) [13].

According to SEM pictures of films EG1, EGA1, and EGC1 shown in Figure 1a,e,i, with the glycol content as 0.3 g, the crosslinked films maintained the same two-phase asymmetric microstructure morphology as compared to films without crosslinking in previous studies [13,14]. Therefore, all the complex films can be simplified to a two-layer structured film as shown in Figure 2, with a porous CEO-rich layer and a compact polymer-rich layer. In Figure 2, *d* is the total thickness of film; *D* is the equivalent diffusion coefficient of CEOs diffused through both the two layers; the thickness of the CEO-rich layer is *d*_1_; and the equivalent diffusion coefficient of CEOs diffused through this layer is *D*_1_. Similarly, the thickness of the polymer-rich layer is *d*_2_, and the equivalent diffusion coefficient of CEOs diffused through this layer is *D*_2_. It is clear that *d* = *d*_1_ + *d*_2_, *D* = *D*−T, *D*_1_ = *D*−B [13].

Thus, the relationship between *D* and both *D*_1_ and *D*_2_ can be simplified from Equation (5) to Equation (6):

(6) $$d/D=d_{1}/D_{1}+d_{2}/D_{2}$$

Then, the relationship between *D*_1_ and *D*_2_ can be transformed from Equation (6) to Equation (7):

(7) $$D_{1}/D_{2}=\left(D_{1}/D-d_{1}/d\right)/\left(1-d_{1}/d\right)$$

Table 2 shows little difference between *D*_1_/*D*_2_ of films G, GA, and GC, which means to catch up to the significant variation of *D*_1_, *D*_2_ should alter at the same rate with *D*_1_ of each film, respectively. The reason for causing a significant difference between *D*_1_ of films G, GA, and GC was assumed to be the different electronical interaction of the emulsifier composition in previous studies. Therefore, *D*_2_ should also be influenced by the electronical interaction of the emulsifier composition. Hence, the polymer-rich layer must be composed of not only SA but also emulsifier chains. Likewise, the CEO-rich layer includes not only the CEO and emulsifier composition but also SA chains. Similar molecular interactions bring out a similar environment for molecular thermal motion, thus allowing for a correlation between *D*_1_ and *D*_2_.

### 3.2. Film Thickness and Mechanical Properties

The films obtained in this study are with good uniformity and peeling facility, similar to that of the previous studies. Table 3 shows that film thickness increases with the increase of glycerol content. This is because the area of film is fixed during the manufacturing process, which leads to the increase of free volume between chains caused by glycerol, which can only increase along the thickness direction.

Table 3 also shows that TS values of all the films are significantly enhanced by crosslinking, with an average of about 17 MPa (films without being crosslinked) [13] increasing to about 38 MPa (crosslinked films). Similar to the films G, GA, and GC without being crosslinked in previous work, the difference of TS among crosslinked EG, EGA, and EGC is still not significant. This might be determined by the crosslinking mechanism. Calcium ion forms a calcium-binding bridge by connecting the carboxyl groups between two SA molecule chains [17] to enhance the TS of the membrane, which has little to do with emulsifiers. For films EG, EGA, and EGC, TS decreased significantly with the increase of glycerol content, which might be because that glycerol increases the chain spacing and reduces the intermolecular force [18].

The elongation at break presented in Table 3 shows that the crosslinking process decreased the *E* of the films, which might be because of the decreased mobility of chain segments [19]. In contrary to crosslinking, the increased glycerol content increased *E* by raising the mobility of chain segments [20]. Moreover, *E* of films EGA and EGC is significantly higher than that of EG, which may be due to the better flexibility of acacia gum chain and more hydroxyl groups on the Arabian gum and carboxymethyl cellulose sodium polysaccharide chains. More hydroxyl groups would adsorb more water molecules, which increases the mobility of chain segments and increases *E*.

### 3.3. Influence of Glycol and Ethanol on D

**Figure 3:**
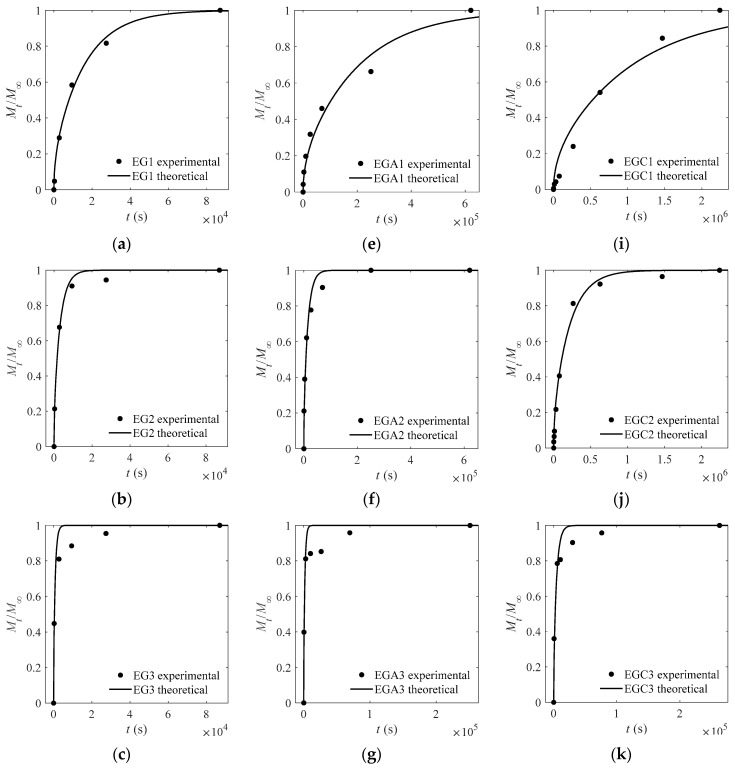

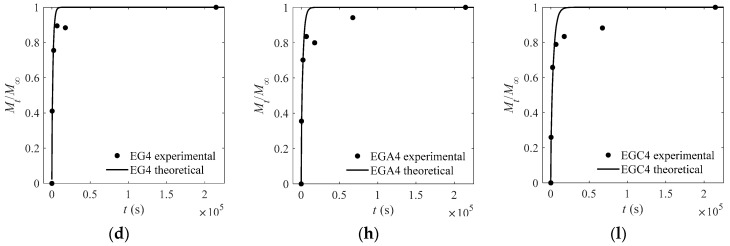
Experimental and theoretical values of one-way release tests of cinnamon essential oils (CEOs) released from films EG1–EG4 (**a**–**d**), EGA1–EGA4 (**e**–**h**), and EGC1–EGC4 (**i**–**l**) to absolute ethanol at 4 °C.

**Figure 4:**
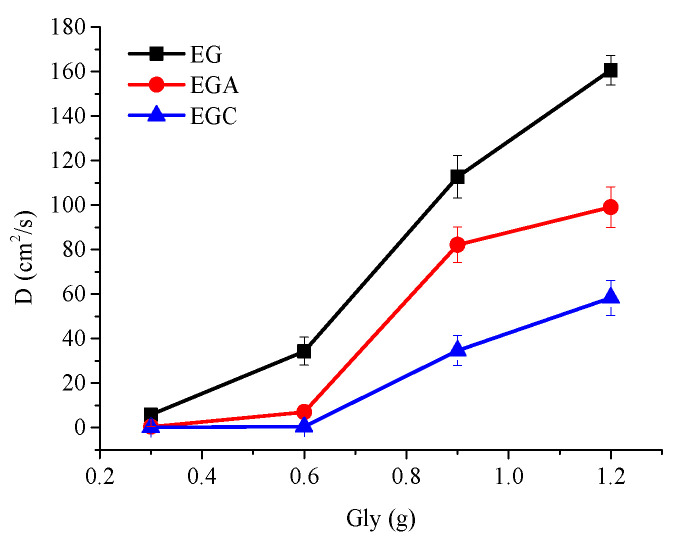
Alteration of diffusion coefficient (*D*) values of CEOs released from films EG, EGA, and EGC to ethanol with the increase of glycerol from 0.3 to 1.2 mL.

**Figure 5:**
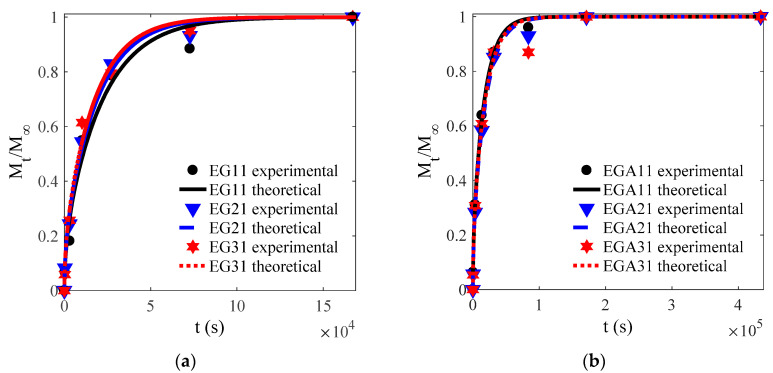

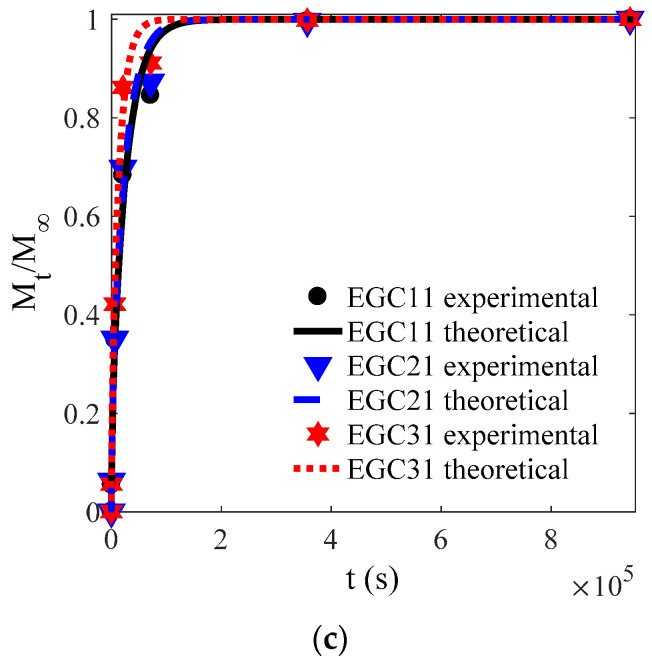
Experimental and theoretical values of one-way release tests of CEOs released from films EG (**a**), EGA (**b**), EGC (**c**) to water at 4 °C.

**Figure 6:**
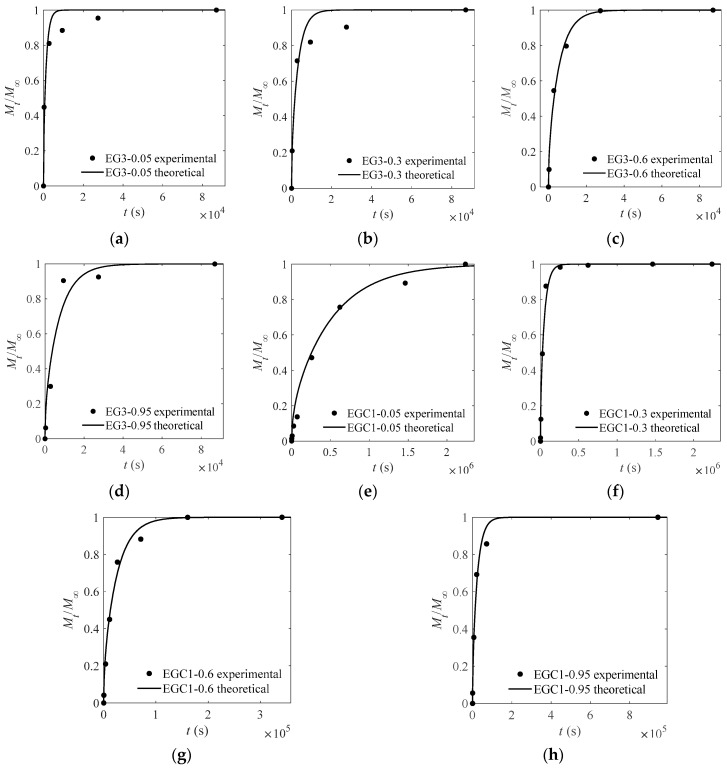
Experimental and theoretical values of one-way release tests of CEOs released at 4 °C from films EG3 (**a**–**d**) and EGC1 (**e**–**h**) to aqueous ethanol with water content of 5%, 30%, 60%, and 95%.

The experimental and theoretical values of the CEO-release proportion out of each film are depicted in Figure 3, Figure 4, Figure 5 and Figure 6 except Figure 4. The goodness of fits is evaluated by means of the root mean square error (RMSE) that are listed in Table 4, Table 5 and Table 6. As can be inferred from the data presented in all the above figures and tables, the model satisfactorily fits the experimental data, suggesting that the adopted model can be used to obtain useful information on the mechanism of CEO release from the crosslinked SA matrices.

Since ethanol is not soluble with the polymer chains, ethanol has no significant effect on the force field formed by the atoms of the polymer chains, and the film does not swell. With solvent ethanol contacting with film, the ethanol molecule enters the film surface through the pores among polymer chains by osmosis. Moreover, because glycerol and water molecules are both soluble in ethanol, the room that is occupied by the glycerol and water well dispersed in the film works as pores or free volume for ethanol molecule. More free volume brings out easier diffusion. Likewise, because CEO is soluble in ethanol, CEOs diffused more easily in the film where ethanol is distributed. Therefore, ethanol acts as an extraction promoting the CEO diffusion.

Specifically, ethanol molecules diffuse firstly from one boundary of the film contacting the solvent through the polymer-rich layer by (1) directly entering pores, (2) dissolving in free water, and (3) dissolving in glycerol among polymer chains. Secondly, reaching the CEO-rich layer, ethanol molecules diffuse in the same way to the interior of the microspheres and dissolve CEOs. Thirdly, ethanol molecules diffuse to the other boundary of film contacting with barrier layers. Unable to penetrate the membrane, ethanol molecules start to move backward. Simultaneously, dissolved CEOs will move out of the film with ethanol, and so do the water and glycerol molecules that are dissolved in ethanol.

The calculated *D* values listed in Table 4 show that for CEOs diffused from films to absolute ethanol, the *D* values increased with a glycerin content increase in all the films. This may be due to two reasons. (1) With solubility among CEOs, ethanol, and glycerin, glycerin itself acts as free volume. More glycerin means more free volume, which increases the space between chains and weakens the interactions among atoms of chains, thus bringing out larger *D* values. (2) Addition of glycerol content as plasticizer increases the space between molecular chains. Meanwhile, with strong hygroscopicity, more glycerol means higher moisture content, and the water molecules also play a similar plasticizing effect as glycerol [21]. Thus, this improves the mobility of the molecular chain segments, reduces the energy required for free volume redistribution, increases the generation frequency of CEOs transition channels, and promotes the diffusion of CEO molecules.

Figure 4 shows that with the increase of glycerol content (0.3, 0.6, 0.9, 1.2 mL), the *D* values of CEOs diffused in films EG, EGA, and EGC showed a similar growth pattern. All of the *D* values increased by small degrees, by a large margin, and in small amounts in the range of 0.3–0.6 mL, 0.6–0.9 mL, and 0.9–1.2 mL, respectively. This may be caused by different distributions of glycerol and the different interactions between chains.

During the increase of glycerol content from 0.3 to 0.6 mL, glycerol might distribute mostly in the original interchain pores, that is, most of the glycerol volume replaces the pore volume without increasing extra spacing between chains. Therefore, the total equivalent free volume in the film does not significantly increase. Glycerol only slightly improves the mobility of molecular segments. Thus, the decrease of free volume redistribution energy is small, and the increase of *D* is small.

In the range of 0.6–0.9 mL, glycerol might distribute more in the extra space produced by increasing spacing between chains. Thus, the total equivalent free volume in the film increases. Moreover, in this range of chain spacing, the intermolecular force decreases rapidly with the increase of spacing. Therefore, the energy required for free volume redistribution reduces greatly, that is, the formation frequency of diffusion channel is greatly increased. Thus, the increase of *D* is large.

In the range of 0.9–1.2 mL, glycerol might as well distribute in the extra space produced by increasing more spacing between chains, which means the total equivalent free volume in the film is further increased and the wall diameter of the diffusion channel is larger. However, in this range of spacing, the intermolecular force decreases slowly with the increase of spacing. Therefore, the energy required for free volume redistribution decreases slightly. Thus, the increase of *D* is small.

In conclusion, the size and distribution of diffusion channels and the intermolecular forces jointly determine the diffusion ability of molecules.

With the increase of glycerol content, the *D* values of CEOs diffused in the film always follow the relationship of *D*_EG_ > *D*_EGA_ > *D*_EGC_. This may be due to the fact that the electrostatic interaction between chains conform to the relationship of GC > GA > G, as discussed in previous work [13]. Furthermore, the *D* values of external crosslinked films are significantly smaller than the *D* values of films without the crosslinking process [13]. It might be the bridging effect that emerged by electrostatic interaction between the –COO^−^ of sodium alginate chains and the Ca^2+^ that increases the interaction between molecular chains [22]. In addition, since the electrostatic interactions enhanced by calcium ion crosslinking are between sodium alginate molecular chains, the ordering of electrostatic interactions between the emulsifier combinations are not affected.

With either the emulsifier combination or the crosslinking process by calcium ions, the mechanism for them to affect CEO diffusion is both through altering the electrostatic interaction between the molecular chains. Higher electrostatic interaction increases the binding effect of chains on CEO molecules and reduces free volume. Therefore, chain segments move more difficultly, and the energy needed for redistribution of free volume increases. Thus, it is more difficult to form a diffusion channel which is not conducive to the diffusion of CEO molecules, and which brings out smaller *D*.

Moreover, it was found that the *D* values of CEOs diffused to absolute ethanol through film EG1 and GA were very close. This might be due to the fact that the electrostatic interaction between –COO^−^ of sodium alginate chains and Ca^2+^ is likely to be the same as that between –COO^−^ of acacia gum and –NH_3_^+^ of the gelatin chain. The *D* values of CEOs diffused to absolute ethanol through films EGA1 and GC were very close. This might be due to the fact that the electrostatic interaction between –COO^−^ of sodium alginate chains and Ca^2+^ and that between –COO^−^ of acacia gum and –NH_3_^+^ of the gelatin chain is likely to be the same as that between –COO^−^ of sodium carboxymethyl cellulose and –NH_3_^+^ of the gelatin chain.

The results also show that the *D* values of CEOs diffused from films EG1 and EGA2 to absolute ethanol are very close. This might be because the electrostatic interaction between –COO^−^ of acacia gum and –NH_3_^+^ of gelatin chain decreased *D* by the same amount with the increment of *D* promoted by 0.3 mL of glycerol in the range of 0.3–0.6 mL. Similarly, the *D* values of CEOs diffused from films EG2 and EGC3 to absolute ethanol are very close. This might be because the electrostatic interaction between –COO^−^ of sodium carboxymethyl cellulose and –NH_3_^+^ of the gelatin chain decreased *D* by the same amount with the increment of *D* promoted by 0.3 mL of glycerol in the range of 0.6–0.9 mL. Moreover, *D* values of CEOs diffused from films EGA1 and EGC2 to absolute ethanol are very close. This might be because the electrostatic interaction between –COO^−^ of sodium carboxymethyl cellulose and –NH_3_^+^ of the gelatin chain minus that between –COO^−^ of acacia gum and –NH_3_^+^ of the gelatin chain decreased *D* by the same amount with the increment of *D* promoted by 0.3 mL of glycerol in the range of 0.3–0.6 mL.

In addition, in the range of 0.3–0.6 mL, the increment of *D*_EGA_ and *D*_EGC_ per unit glycerol is significantly smaller than that of *D*_EG_. This may be due to the stronger electrostatic interaction between the chains in films EGA and EGC, which squeezes more glycerol into the holes originally existing between chains. Moreover, there are more hydroxyl groups in acacia gum and sodium carboxymethyl cellulose molecular chains than in gelatin chains. Since the hydroxyl groups have stronger adsorption on glycerol, glycerol might distribute more finely and stably in the holes between chains, thus reducing the influence on diffusion.

### 3.4. Influence of Water on D

With the solvent water contacting films, water molecules permeate the film surface through osmosis. Because the polymer chains composing films are rich in hydroxyl groups that have good compatibility with water, water molecules firstly form binding water with the hydroxyl groups in the chain by hydrogen bonding. Then, after all the available binding formed, extra water molecules distribute among chains as free water and swell the film. Thus, the spacing between chains increase, the atomic force field is weakened, and the blocking effect of atomic force field on the diffusion of CEOs is reduced, which is beneficial to the diffusion of CEOs. However, because CEO is slightly soluble in water, the place where water molecules distribute is similar to a barrier. Thus, water itself plays a blocking role in the diffusion of CEOs. Overall, the *D* values of CEOs diffused from films to water are determined by two kinds of blocking effects: the reduced blocking effect of atomic force field of polymer chain and the blocking effect of water.

The calculated *D* values listed in Table 5 show that the effect of either electrostatic interaction (emulsifier combination, crosslinking process), or the amount of free volume and intermolecular force (plasticizer content) on *D* values of CEOs diffused from films to water are not significant. *D* values are almost all in the order of magnitude of 10^−10^ cm^2^/s. This shows that the influence of swelling effect on the molecular force field of the film is far greater than other factors, which dramatically reduces the difference between films. This may be because the swelling effect greatly increases the spacing between molecular chains, which reduces the intermolecular forces of different films to a similar small value. In other words, the blocking effect of a high molecular chain force field in different films is similar. Moreover, water has an identity-blocking effect on the diffusion of CEOs through different films. Therefore, the diffusion ability of CEOs diffused from different films to water is close, and there is no significant difference in diffusion coefficient.

### 3.5. Influence of Aqueous Ethanol on D

For the solvent containing both ethanol and water, i.e., aqueous ethanol, the influence on *D* consists of the blocking effect of polymer chains (reduced by water), the blocking effect of water, and the extraction-promoting effect by ethanol. Here, the one that plays a leading role determines the diffusion ability of CEO.

**Figure 7:**
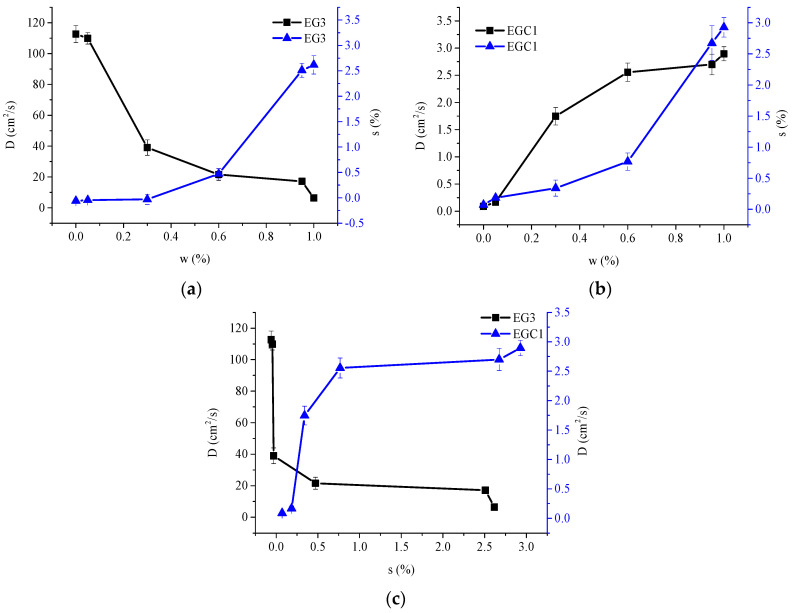
*D* values of CEOs released from films EG3 (**a**) and EGC1 (**b**) to aqueous ethanol of *w* = 0, 0.05, 0.3, 0.6, 0.95 and 1, respectively; *s* at equilibrium of films EG3 (**a**) and EGC1 (**b**) contacting aqueous ethanol of *w* = 0, 0.05, 0.3, 0.6, 0.95 and 1, respectively; *D* values at different *s* of CEOs released from films EG3 and EGC1 to aqueous ethanol (**c**), respectively.

Table 6 shows that for those *D* values of CEOs diffused to ethanol that are much greater or less (two orders of magnitude, for instance) than those which diffuse to water, the diffusion of CEOs in the film is very sensitive to water. As shown in Figure 7a, *D* can be greatly changed by a low moisture content and small swelling rate. For instance, for film EG3, *D* decreases dramatically in the range of a low percentage of moisture in the solvent (*w* = 0 to 0.3) and film (*s* = −0.06 to −0.03). This might be due to the long spacing between polymer chains, which has already made the film reach the range of weak and slow change of a molecular force field (as discussed in Section 3.3). Thus, the promoting effect by swelling and ethanol extraction is not significant. On the contrary, with the increase of percentage of moisture in the solvent, the partition coefficient decreased greatly, and the blocking effect of water plays a leading role, hence greatly reducing the *D* of CEOs.

In another case, for film EGC1, as shown in Figure 7b, *D* increased dramatically in the range of low percentage of moisture in the solvent (*w* = 0–0.3) and in film (*s* = 0.07–0.34). This might be because the short spacing between polymer chains (which appeared by strong molecular force field as discussed in Section 3.3) is significantly enlarged by the water swelling effect. Thus, the reduced blocking effect of polymer chains by water plays a leading role, hence greatly increasing the *D* of CEO.

Figure 7 also shows that in the range of *w* = 0–0.6, the swelling ratio of two films increased to 0.47 (EG3) and 0.77 (EGC1), respectively. Both the *D* values varied considerably first and then slightly. In the range of *w* = 0.6–1, the swelling ratio of two films increased to 2.6 (EG3) and 2.9 (EGC1), respectively, while both the *D* values changed only slightly. The results implicate that (1) for film EG3, the partition coefficient might decrease slightly when *w* > 0.6; and (2) for film EGC1, when *s* > 0.77, the spacing between chains might have already made the film reach the range of weak and slow change of the molecular force field as described before. Therefore, the *D* values of CEOs varied slightly with vigorously increased swelling rates. In addition, the swelling rate of the film EGC1 is higher than that of film EG3, which may be due to the retention of more water molecules by more hydroxyl groups on sodium carboxymethyl cellulose in film EGC1.

## 4. Conclusions

Overall, the calcium alginate based controlled release films fabricated in this paper obtained moderate mechanical properties. Glycerol plays a promoting role for CEOs released from films to ethanol. With the increase of glycerol content, *D* values of CEOs diffused to ethanol firstly increased slowly (0.3–0.6 mL), then vigorously (0.6–0.9 mL), and then mildly (0.9–1.2 mL). With a strong swelling effect of water on films, the *D* values of CEOs diffused to water are all in the order of magnitude of 10^−10^ cm^2^/s, eliminating the distinct difference of the *D* values of CEOs diffused to ethanol. The *D* values of CEOs diffused from films EG3 and EGC1 to aqueous ethanol altered enormously at a small moisture percentage (*w* = 0.3) and a low swelling rate. Then, in the range of *w* = 0.3–1, the *D* values of CEOs diffused to aqueous ethanol varied vigorously at first and then mildly. All of the above results indicating that, considering the influence of the osmotic solvent, the size and distribution of free volume in the system (polymer + diffusive substance + solvents), the intermolecular interaction, and the partition coefficient of the solvents jointly determined the diffusion ability of the molecules. Moreover, the pairs of *D*_EG_ and *D*_GA_, *D*_GC_ and *D*_EGA_, *D*_EG1_ and *D*_EGA2_, *D*_EGA1_ and *D*_EGC2_, and *D*_EG2_ and *D*_EGC3_ have values that are very close to one another, indicating that different kinds of interactions between different groups may have the same effect on the diffusion ability of molecules. The correlation between *D*_1_ and *D*_2_ indicates that in the polymer-rich layer of two-layer structured films, polymeric emulsifier chains also exists. Based on all the theoretical analysis and experimental data presented in this paper, we will further study the diffusion mechanism through molecular dynamic simulation in future works. Understanding the microscopic mechanism of active substance release through bio-based film would help to guide the design of controlled release food packaging for better food protection.

## Figures and Tables

**Table 1:** Formulation of the films.

Film Samples	Film Compositions	Anhydrous Composition Contents (g)
EG*_kf_*	SA + (G_0_ + CEO) + Gly	0.6; 0.25; 0.5; 0.3*k*
EGA*_kf_*	SA + (G_0_ + A_0_ + CEO) + Gly	0.6; 0.225; 0.025; 0.5; 0.3*k*
EGC*_kf_*	SA + (G_0_ + C_0_ + CEO) + Gly	0.6; 0.225; 0.025; 0.5; 0.3*k*

The parameter *k* equals to 1, 2, 3, and 4, respectively, representing different content of Gly (0.3, 0.6, 0.9, and 1.2 g). The parameter *f* represents different FSSs: For absolute ethanol, *f* equals to null; for water, *f* equals to 1. For example, EG1 and EG11 represent films with 0.3 g Gly and FSS was absolute ethanol and water, respectively.

**Table 2:** Relationship between diffusion coefficient of two layers (*D*_1_ and *D*_2_ [13]) of film G, GA, and GC.

Film Samples	*D* (cm^2^/s)	*D*_1_ (cm^2^/s)	*D*_1_/*D*	*d*_1_/*d*	*D*_1_/*D*_2_
G	2.000 × 10^−9^	8.064 × 10^−9^	4.032	0.75	13.13
GA	6.680 × 10^−10^	2.950 × 10^−9^	4.416	0.73	13.47
GC	4.140 × 10^−11^	1.760 × 10^−10^	4.251	0.74	13.27

The values of *D* and *D*_1_ of each film were cited from previous work; *d*_1_/*d* of G, GA, and GC were obtained from Figure 2a–c, respectively. *D*_1_/*D*_2_ was calculated by Equation (5).

**Table 3:** Film thickness and mechanical properties of films.

Film Samples	Thickness (mm)	(s.d.)	*TS*/MPa	(s.d.)	*E*/%	(s.d.)
EG1	0.039 ^a^	0.003	40.82 ^a^	4.344	2.888 ^a^	0.141
EG2	0.044 ^b^	0.001	27.44 ^b^	3.672	9.760 ^a^	5.794
EG3	0.047 ^b^	0.002	16.73 ^c^	1.646	21.44 ^b^	5.226
EGA1	0.042 ^a^	0.003	34.83 ^a^	3.200	15.01 ^c^	4.487
EGA2	0.042 ^a^	0.006	26.83 ^b^	2.103	18.05 ^c^	8.403
EGA3	0.047 ^a^	0.008	19.43 ^c^	4.183	36.83 ^d^	8.832
EGC1	0.046 ^a^	0.002	38.34 ^a^	3.295	12.92 ^c^	4.225
EGC2	0.052 ^bc^	0.001	24.34 ^b^	4.264	28.10 ^b^	3.605
EGC3	0.057 ^cd^	0.004	15.85 ^c^	2.284	42.54 ^e^	8.137

Means followed by the same letter in a column are not significantly different from each other at *p* < 0.05.

**Table 4:** Diffusion coefficients of one-way release of CEOs released from films to absolute ethanol at 4 °C.

Film Samples	Thickness (mm)	*D* (cm^2^/s)	RMSE
EG1	0.048	5.800 × 10^−10^ ^a^	0.03248
EG2	0.053	3.433 × 10^−9^ ^b^	0.03436
EG3	0.062	1.096 × 10^−8^ ^c^	0.09119
EG4	0.079	1.606 × 10^−8^ ^d^	0.07104
EGA1	0.047	4.200 × 10^−11^ ^e^	0.05190
EGA2	0.051	7.000 × 10^−10^ ^a^	0.06900
EGA3	0.059	8.216 × 10^−9^ ^f^	0.09958
EGA4	0.076	9.900 × 10^−9^ ^g^	0.1029
EGC1	0.049	8.800 × 10^−12^ ^h^	0.07831
EGC2	0.050	4.510 × 10^−11^ ^e^	0.04025
EGC3	0.062	3.340 × 10^−9^ ^b^	0.07512
EGC4	0.072	5.938 × 10^−9^ ^i^	0.09671

Means followed by the same letter in a column are not significantly different from each other at *p* < 0.05.

**Table 5:** Diffusion coefficients of one-way release of CEOs released from films to water at 4 °C.

Film Samples	Thickness (mm)	*D* ((cm^2^/s) × 10^−10^)	RMSE
EG11	0.047	4.050 ^a^	0.05661
EG21	0.049	5.100 ^a^	0.03139
EG31	0.053	6.354 ^a^	0.04163
EGA11	0.044	4.500 ^a^	0.01860
EGA21	0.050	5.085 ^a^	0.02774
EGA31	0.063	8.500 ^a^	0.04937
EGC11	0.046	2.896 ^a^	0.06030
EGC21	0.052	4.400 ^a^	0.04030
EGC31	0.060	9.988 ^a^	0.04996

Means followed by the same letter in a column are not significantly different from each other at *p* < 0.05.

**Table 6:** Diffusion coefficients of one-way release of CEOs from films to aqueous ethanol at 4 °C.

Film Samples	*w*	Thickness (mm)	*D* (cm^2^/s)	RMSE
EG3	0.00	0.062	1.127 × 10^−8 a^	0.08408
EG3-0.05	0.05	0.062	1.099 × 10^−8 a^	0.08413
EG3-0.30	0.30	0.057	3.900 × 10^−9 b^	0.07883
EG3-0.60	0.60	0.056	2.156 × 10^−9 c^	0.04087
EG3-0.95	0.95	0.057	1.712 × 10^−9 c^	0.09820
EG31	1.00	0.053	6.354 × 10^−10 d^	0.04163
EGC1	0.00	0.049	8.800 × 10^−12 e^	0.07830
EGC1-0.05	0.05	0.047	1.670 × 10^−11 f^	0.05916
EGC1-0.30	0.30	0.048	1.747 × 10^−10 d^	0.05616
EGC1-0.60	0.60	0.041	2.556 × 10^−10 d^	0.04232
EGC1-0.95	0.95	0.041	2.700 × 10^−10 d^	0.04515
EGC11	1.00	0.046	2.896 × 10^−10 d^	0.06030

Means followed by the same letter in a column are not significantly different from each other at *p* < 0.05.
